# *Pseudomonas putida* Represses JA- and SA-Mediated Defense Pathways in Rice and Promotes an Alternative Defense Mechanism Possibly through ABA Signaling

**DOI:** 10.3390/plants9121641

**Published:** 2020-11-24

**Authors:** Rui Wang, Hai-Lin Wang, Rui-Ping Tang, Meng-Ying Sun, Tang-Min Chen, Xu-Chu Duan, Xiao-Feng Lu, Dong Liu, Xin-Chi Shi, Pedro Laborda, Su-Yan Wang

**Affiliations:** School of Life Sciences, Nantong University, Nantong 226019, China; 1709011048@stmail.ntu.edu.cn (R.W.); 1709011051@stmail.ntu.edu.cn (H.-L.W.); 1909110173@stmail.ntu.edu.cn (R.-P.T.); 1909110216@stmail.ntu.edu.cn (M.-Y.S.); 1909110125@stmail.ntu.edu.cn (T.-M.C.); dxd2002sk@ntu.edu.cn (X.-C.D.); luxf@ntu.edu.cn (X.-F.L.); tom@ntu.edu.cn (D.L.)

**Keywords:** *Oryza sativa*, plant growth-promoting rhizobacteria, plant–rhizobacteria interaction, plant defense, abscisic acid

## Abstract

The signaling pathways induced by *Pseudomonas putida* in rice plants at the early plant–rhizobacteria interaction stages, with and without inoculation of *Xanthomonas oryzae* pv. *oryzae*, were studied. In the absence of pathogen, *P. putida* reduced ethylene (ET) production, and promoted root and stem elongation. Interestingly, gene *OsHDA702*, which plays an important role in root formation, was found significantly up-regulated in the presence of the rhizobacterium. Although *X. oryzae* pv. *oryzae* inoculation enhanced ET production in rice plants, *P. putida* treatment repressed ET-, jasmonic acid (JA)- and salicylic acid (SA)-mediated defense pathways, and induced the biosynthesis of abscisic acid (ABA), and the overexpression of *OsHDA705* and some pathogenesis-related proteins (PRs), which in turn increased the susceptibility of the rice plants against the pathogen. Collectively, this is the first work on the defense signaling induced by plant growth-promoting rhizobacteria in plants at the early interaction stages, and suggests that rhizobacteria stimulate an alternative defense mechanism in plants based on ABA accumulation and *OsHDA705* signaling.

## 1. Introduction

Phytohormones salicylic acid (SA), ethylene (ET), jasmonic acid (JA), abscisic acid (ABA), indole-3-acetic acid (IAA), and gibberellic acid (GA) are known to modulate plant growth and defense responses [1,2]. ET and ABA mainly function as plant growth inhibitors, whereas the accumulation of GA and IAA has been related in several occasions to plant growth promotion [3]. SA signaling positively regulates plant defense against biotrophic pathogens, whereas ET/JA pathways are commonly required for resistance to necrotrophic pathogens and to herbivorous pests. Increased ABA levels have been correlated with enhanced susceptibility to biotic pathogens due to ABA acts synergistically with ET-mediated signaling [4,5]. In contrast, ABA is able to enhance plant tolerance to abiotic stresses, such as drought, salinity, or extreme temperatures [6].

The toxic effects of some traditional pesticides to human health and environment have stimulated the development of new environmental-friendly strategies [7,8,9]. In this sense, induced resistance strategies have been postulated as an alternative in modern and sustainable agriculture. Two main mechanisms of induced resistance in plants against pathogens have been described to date: Systemic acquired resistance (SAR) and induced systemic resistance (ISR) [10,11]. SAR is induced via infection by a virulent pathogen, whereas the presence of a plant growth-promoting rhizobacterium is required for ISR. Recent reports have indicated that the ISR effects are prolonged during several days [12].

Glick and co-workers established that growth-promoting bacteria are able to colonize plant roots, inducing the release of 1-amino-1-carboxylic acid (ACC), the main precursor of ET, from the root cells into the medium [13,14,15]. The resulting low concentrations of ACC inside cells decrease the plant ET levels, reducing ET-related stress and inducing plant growth promotion [16,17]. Most rhizobacterial strains are able to express ACC deaminase, which is able to transform the released ACC into ammonia and 2-oxobutanoic acid. Contradictorily, ISR mechanism, which is also activated in the presence of growth-promoting rhizobacteria, relies on the induction of the ET signaling pathway [18]. In order to explain how rhizobacteria can simultaneously produce both effects, several research groups have explored the metabolic regulations induced by rhizobacteria on plants during recent years. In this sense, some reports have indicated that rhizobacteria are able to modify plant ET levels, decreasing ET production at the early plant–bacteria interaction stages, and enhancing ET levels at the late interaction stages [19,20]. Although the enhanced disease resistance at the late plant–bacteria interaction stages is dependent on the up-regulation of the ET and JA signaling pathways (ISR mechanism), the key signaling pathways involved in defense response at the early plant–bacteria interaction stages remain unclear [21].

*Pseudomonas putida* is a well-known plant growth-promoting rhizobacterium thoroughly used as standard for the study of plant–bacteria interactions [22]. *Xanthomonas oryzae* pv. *oryzae* is a hazardous bacterial pathogen able to cause bacterial blight disease on rice [23]. This pathogen produces yellow lesions on the rice leaves, inhibiting the photosynthesis and reducing the production and quality of the rice crops. In this work, the interaction between *P. putida*, and rice plants, *Oryza sativa* cv. Nipponbare, has been examined in order to better understand the signaling pathways involved in growth promotion and disease resistance at the early plant–rhizobacteria interaction stages. Interestingly, it was found that *P. putida* colonized rice roots, reduced the ET emission and promoted the accumulation of hormone ABA in response to *X. oryzae* pv. *oryzae*.

## 2. Results

### 2.1. P. putida Can Colonize Rice Roots, and Promotes Root and Stem Elongation

**Figure 1:**
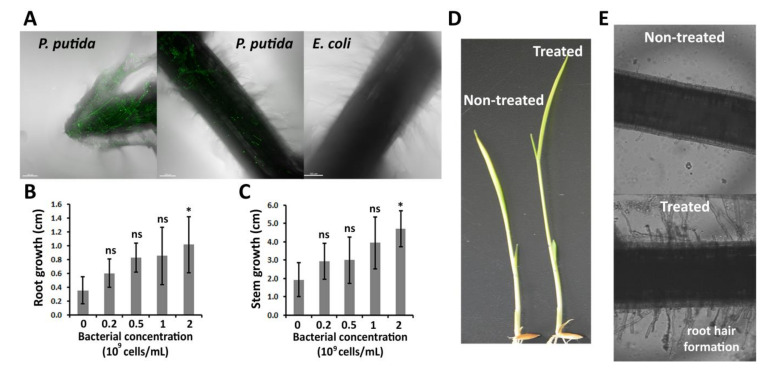
Colonization and growth promotion effects of *Pseudomonas putida* on rice plants. (**A**) *P. putida* transformants colonizing root hair and cap. Scale bar = 100 μm. (**B**) Root and (**C**) stem growth of rice plants in the presence of *P. putida*. The length of roots and stems was measured 2 days after *P. putida* treatment (early interaction stages). (**D**) Images of rice plants treated with 0 and 2 × 10^9^ cells/mL *P. putida*. (**E**) Microscope images showing the root hair formation of rice plants treated with 0 and 2 × 10^9^ cells/mL *P. putida*. The images were taken 2 days after the bacterial treatment. Significance levels at *p* < 0.05 (*) and no significance (ns).

*P. putida* transformants producing green fluorescent protein (GFP) were able to colonize rice roots, whereas *Escherichia coli* transformants, which were used as negative control, could not colonize the roots (Figure 1A). As far as we know, this is the first report of the ability of *P. putida* to colonize rice roots. The green color was mainly observed in the root hair and cap, indicating that *P. putida* preferentially colonizes these parts of the rice root.

As shown in Figure 1B–D, *P. putida* promoted root and stem elongation during the first 48 h after the treatment. The length promotion was proportional to the concentration of bacteria. In this sense, 0.25, 0.5, 1, and 2 × 10^9^ cells/mL *P. putida* increased the stem length by 53%, 56%, 105%, and 145%, respectively. Similarly, the root was 71%, 135%, 143%, and 189% longer in the presence of 0.25, 0.5, 1, and 2 × 10^9^ cells/mL *P. putida*, respectively, in comparison with the root length of the control group, which was performed in the absence of rhizobacterium. By comparing the promotion in root and stem, it can be observed that the effects on root were more obvious that the effects on stem.

Microscope observations indicated that *P. putida*-treated plants showed longer root hair in comparison with the non-treated plants (Figure 1E). This effect was also observed when rice plants were treated with plant growth-promoting rhizobacterium *Lysobacter gummosus* [24].

### 2.2. P. putida Induces ACC Release from Rice Plants into the Medium

**Figure 2:**
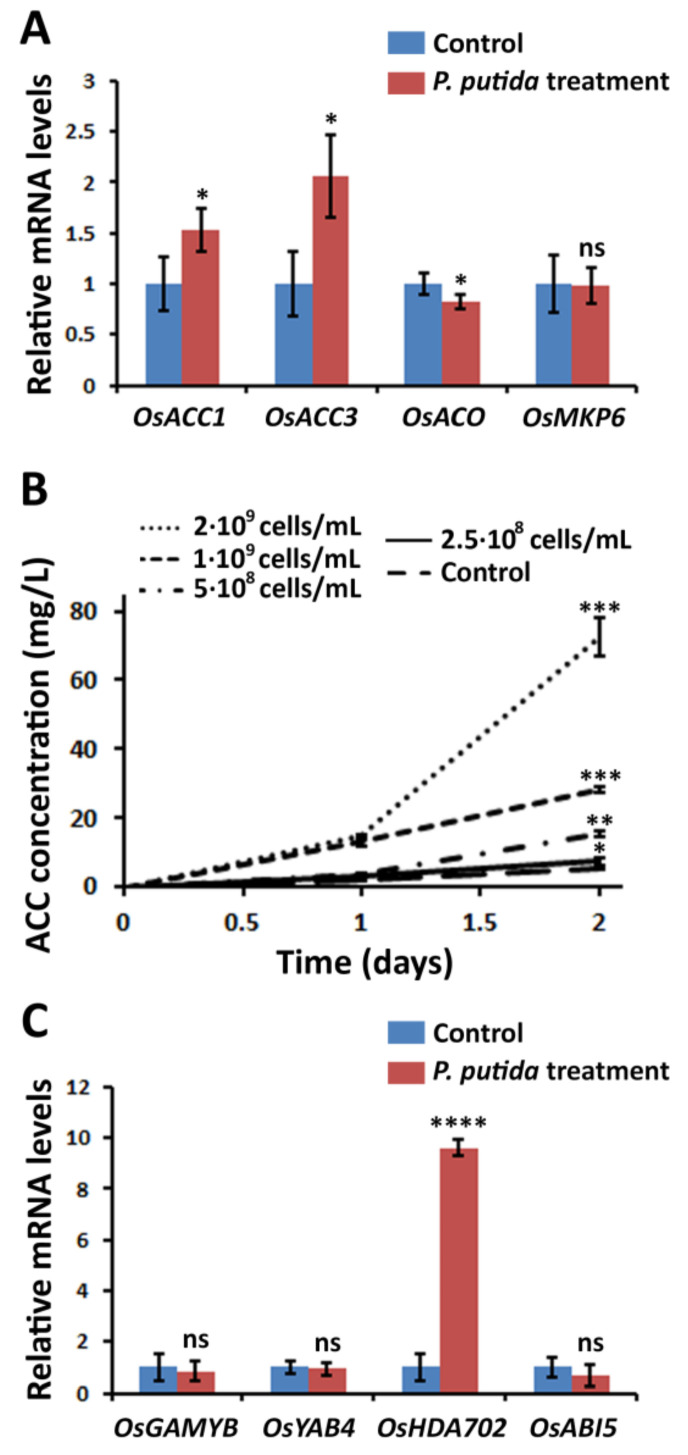
Metabolic alterations produced by *P. putida* in rice plants. (**A**) Relative mRNA levels of ethylene (ET) biosynthesis and signaling genes in the presence and absence of *P. putida*. (**B**) Concentration of 1-amino-1-carboxylic acid (ACC) in rice culture medium in the presence and absence of *P. putida*. (**C**) Relative mRNA levels of gibberellic acid (GA) and abscisic acid (ABA) signaling genes in the presence and absence of *P. putida*. Significance levels at *p* < 0.05 (*), *p* < 0.01 (**), *p* < 0.001 (***), *p* < 0.0001 (****) and no significance (ns).

In order to understand the metabolic alterations involved in the elongation of root and stem, the mRNA levels of relevant genes involved in ET biosynthesis were measured. It was found that *OsACO*, which is involved in the transformation of ACC into ET, was down-regulated in the treated plants (Figure 2A), which indicated that the ET emission was reduced in the presence *P. putida*. In contrast, *OsACC1* and *OsACC3*, which are involved in the transformation of *S*-adenosyl-L-methionine into ACC, were 1.52 and 2.06 times up-regulated in the treated plants. These results suggested that the rice plants are producing more ACC in the presence of *P. putida*; however, this ACC is not transformed into ET.

Interestingly, ACC was detected in the rice culture medium (Figure 2B), indicating that the produced ACC is released from the rice roots into the medium. The concentration of ACC in the medium increased when enhancing the amount of the bacteria. The control plants, which were prepared in the absence of *P. putida*, also released ACC, but in much lower concentration in comparison with the *P. putida*-treated plants, confirming that *P. putida* is inducing the release of ACC. In this sense, the concentration of ACC in the medium of the rice plants treated with 2 × 10^9^ cells/mL was 14 times higher with respect that of the control plants. Although *P. putida* is able to express ACC deaminase, our results indicated that ACC is accumulated in the medium, suggesting that the amount of ACC consumed by the rhizobacteria is lower in comparison with the released ACC.

The expression levels of key genes involved in rice growth were examined (Figure 2C). *OsMPK6* is a mitogen-activated protein kinase that regulates plant height, and has been related to the signaling of different hormones, including ET or ABA. No significant differences were observed in the mRNA levels of *OsMPK6* in the treated and non-treated plants, indicating that this gene does not participate in the elongation effect. Two genes related to GA signaling pathways, including *OsGAMYB* and *OsYAB4*, were studied. The mRNA levels of both genes were not significantly changed in the treated plants, suggesting that GA is not involved in the elongation effect. One gene directly related to ABA signaling was studied, namely *OsABI5*, which is involved in rice development and fertility. No significant change was found in the mRNA level of *OsABI5* in the treated plants, indicating that ABA might not be responsible for the elongation effect. In contrast, histone deacetylase *OsHDA702*, a gene involved in root development that belongs to the signaling pathway of IAA, was 9.6-fold up-regulated in the treated plants in comparison with the non-treated plants. One possible explanation of the observed results might be that the IAA signaling is activated in the presence of *P. putida*, and may play an important role in the observed elongation effect.

### 2.3. P. putida Inhibits ET Biosynthesis in Response to X. oryzae pv. oryzae Causing Bacterial Blight

**Figure 3:**
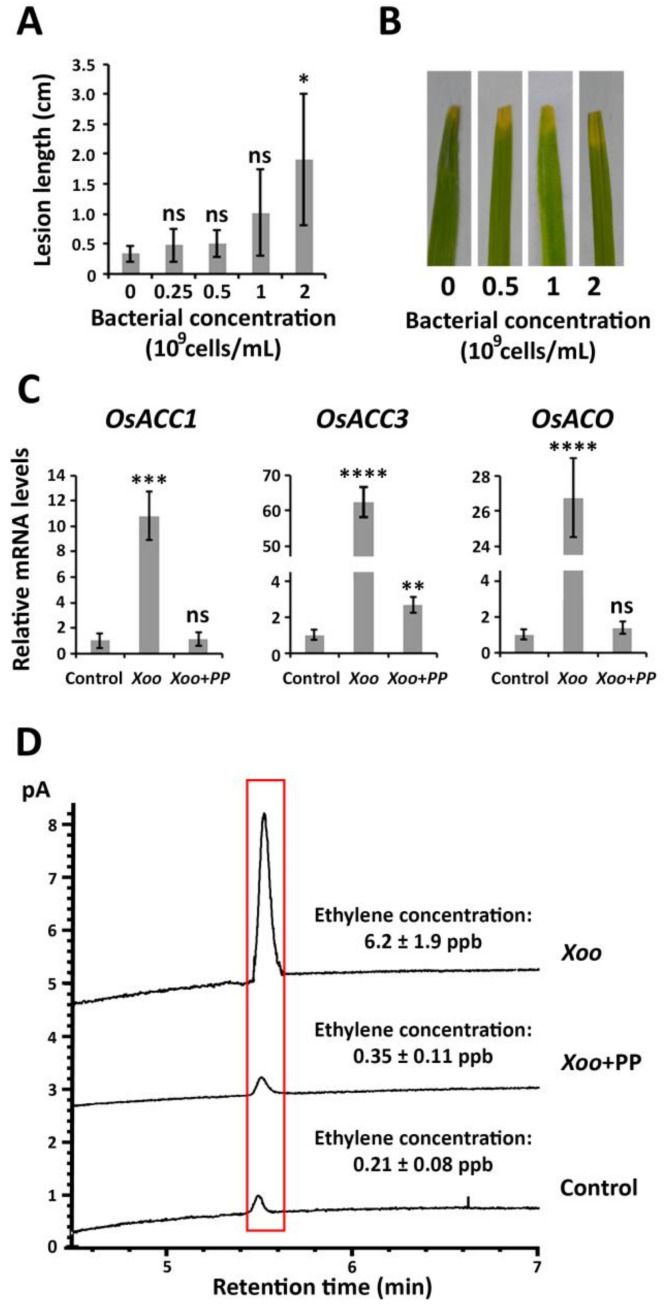
Effects produced by *P. putida* on rice disease resistance and ET biosynthesis after *X. oryzae* pv. *oryzae* inoculation. (**A**) Lesion length produced by *Xanthomonas oryzae* pv. *oryzae* in rice plants. The disease advancement was measured 2 days after inoculation of the pathogen. (**B**) Images showing the pathogen advancement. (**C**) Relative mRNA levels of ET biosynthesis genes *OsACC1*, *OsACC3* and *OsACO* in *X. oryzae* pv. *oryzae*-infected rice plants with and without *P. putida* treatment. The control experiment was performed in the absence of pathogen and rhizobacteria. (**D**) Gas chromatography-based analysis of ET production levels in *X. oryzae* pv. *oryzae*-infected rice plants with and without *P. putida* treatment. Significance levels at *p* < 0.05 (*), *p* < 0.01 (**), *p* < 0.001 (***), *p* < 0.0001 (****) and no significance (ns).

The disease resistance of rice plants against *X. oryzae* pv. *oryzae* was evaluated according to the lesion length caused by the bacterial pathogen, which was measured 48 h after the inoculation (early bacteria–plant interaction stages) (Figure 3A,B). The presence of 0.25, 0.5, 1, and 2 × 10^9^ cells/mL *P. putida* increased the lesion length by 43%, 50%, 205%, and 475%, respectively, in comparison with the control experiment, which was performed in the absence of rhizobacteria. These results indicated that *P. putida* is decreasing the innate disease resistance of the rice plants against *X. oryzae* pv. *oryzae*. The disease incidence was constant, between 80% and 85%, across the different treatment conditions.

In the absence of *P. putida*, ET biosynthesis increased in response to *X. oryzae* pv. *oryzae*. As it can be observed in Figure 3C, the mRNA levels corresponding to *OsACC1*, *OsACC3,* and *OsACO* in the *X. oryzae* pv. *oryzae*-infected plants were 11, 63, and 27 times higher, respectively, in comparison with those detected in the control plants.

In contrast, the mRNA levels of *OsACO* and *OsACC1* remained unchanged in the *X. oryzae* pv. *oryzae*-infected plants under treatment with *P. putida*, whereas the expression of *OsACC3* was only 2.6-fold up-regulated. In order to confirm that *P. putida* is decreasing ET emission after *X. oryzae* pv. *oryzae* infection, the ET levels of the rice plants were measured by gas chromatography. The obtained results indicated that the ET level in the rice plants treated with *P. putida* was 17.7 times lower in comparison with the ET level of the infected rice plants in the absence of rhizobacteria. These results suggested that the rice plants in the absence of *P. putida* activate a mechanism of defense based on ET biosynthesis; however, it appears that the rice plants treated with *P. putida* do not activate an ET-based defense response.

### 2.4. P. putida Activates an Alternative Mechanism of Defense in Rice Plants Possibly Based on ABA Accumulation

**Figure 4:**
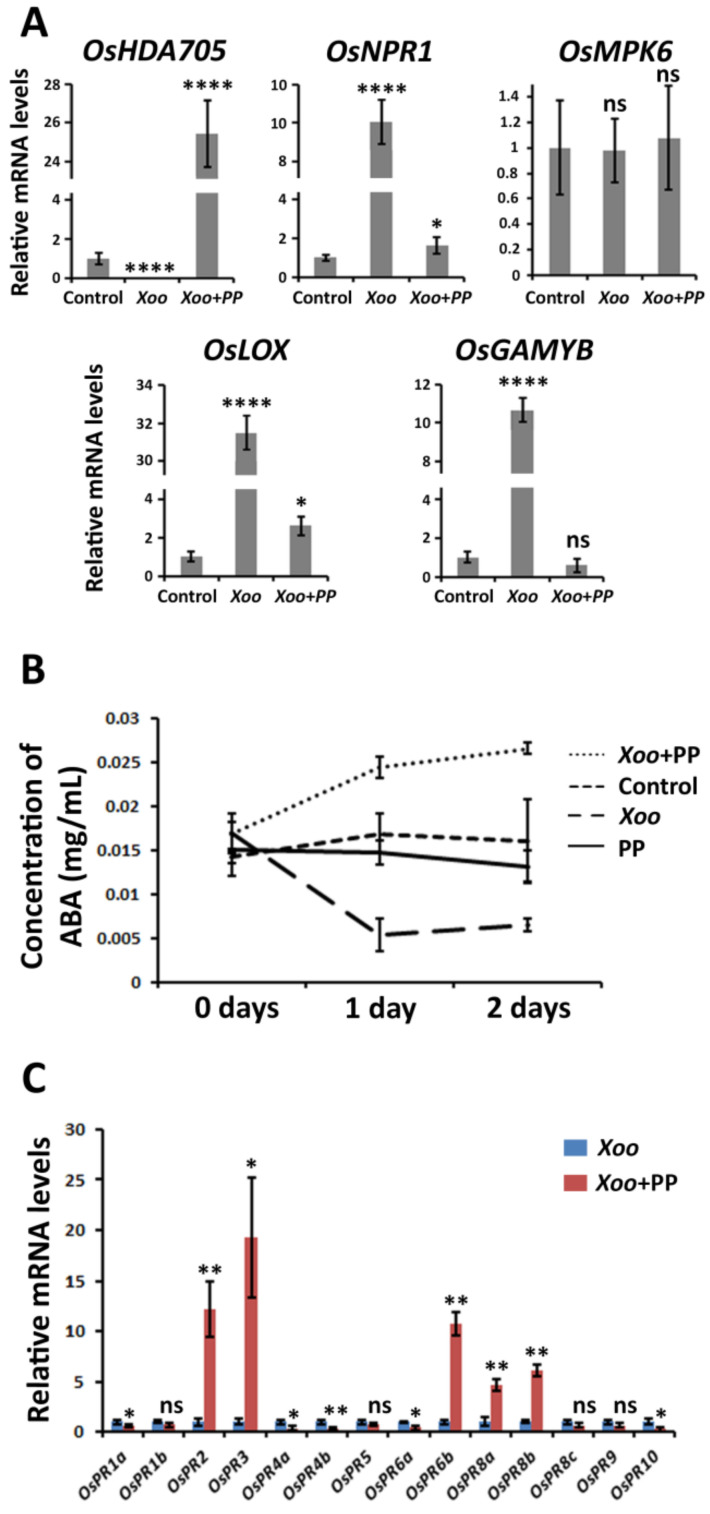
*P. putida* induces an alternative defense mechanism in *X. oryzae* pv. *oryzae*-infected rice plants. (**A**) mRNA levels of relevant signaling genes related to rice defense. RNA extraction from rice leaves was carried out 2 days after pathogen inoculation. (**B**) Concentration of ABA in *X. oryzae* pv. *oryzae*-infected rice plants in the presence and absence of *P. putida*. The control experiment was performed in the absence of pathogen and rhizobacteria. (**C**) mRNA levels of pathogenesis-related proteins (PRs) in *X. oryzae* pv. *oryzae*-infected rice plants in the presence and absence of *P. putida*. Significance levels at *p* < 0.05 (*), *p* < 0.01 (**), *p* < 0.0001 (****) and no significance (ns).

To understand the metabolic pathways involved in the defense of *P. putida*-treated rice against *X. oryzae* pv. *oryzae*, the expression of several genes involved in rice defense was examined (Figure 4A). To achieve this goal, 3 parallel experiments were performed, namely *X. oryzae* pv. *oryzae*-infected plants treated with *P. putida* (*Xoo*+*PP*), *X. oryzae* pv. *oryzae*-infected plants in the absence of *P. putida* (*Xoo*), and plants in the absence of *X. oryzae* pv. *oryzae* and *P. putida* (Control).

*OsMPK6* was unaltered under the 3 treatment conditions. The expression of GA-signaling gene *OsGAMYB* remained unaltered in the *Xoo*+*PP* plants in comparison with the control plants; however, this gene was 10-fold up-regulated in the *Xoo* plants. *OsNPR1* is a gene involved in the signaling of ET and SA in response to pathogen infection. This gene was 10-fold up-regulated in the *Xoo* plants, but only 1.6-fold up-regulated in the *Xoo*+*PP* plants. The mRNA levels of *OsLOX*, which is involved in JA signaling, were 31 times higher in the *Xoo* plants in comparison with those detected in the control plants, and only 2.6 times higher in the *Xoo*+*PP* plants. These results suggest that the GA, ET, and JA signaling pathways are activated in response to the pathogen in the absence of bacteria. However, *P. putida* appears to repress the activation of the usual defense response.

In contrast, *OsHDA705*, a histone deacetylase involved in ABA signaling, was 26-fold up-regulated in the *Xoo*+*PP* plants, and 50-fold down-regulated in the *Xoo* plants. One possible explanation of these results might be that ABA is involved in the defense response of *P. putida*-treated rice plants; however, the down-regulation of *OsHDA705* in the *Xoo* plants seems to indicate that ABA is not involved in the defense response of the rice plants in the absence of rhizobacteria.

In order to confirm that the biosynthesis of ABA is induced in the *Xoo*+*PP* plants, the concentration of ABA in the rice leaves was measured under different treatment conditions (Figure 4B). It was detected that the concentration of ABA remained constant in the *P. putida*-treated plants without *X. oryzae* pv. *oryzae* inoculation (*PP*). On the other hand, the concentration of ABA increased in the *Xoo*+*PP* plants by 80% with respect the concentration in the control plants, whereas the concentration of ABA decreased in the *Xoo* plants by 73%. These results are consistent with the mRNA levels of *OsHDA705* in the *Xoo*+*PP* and *Xoo* plants, and suggest that *P. putida*-treated plants rice plants are possibly activating the biosynthesis of ABA in response to *X. oryzae* pv. *oryzae* infection. The accumulation of ABA in the rice plants was only observed in the presence of the pathogen, but not in the *P. putida*-treated plants in the absence of pathogen infection, suggesting that an external stress is necessary to initiate the accumulation of ABA. This result is consistent with the observed mRNA level of ABA-signaling gene *OsABI5* in *P. putida*-treated plants, which was not affected by *P. putida* (Figure 2C).

### 2.5. P. putida Activates the Expression of Alternative Pathogenesis-Related Genes (PRs)

The expression levels of pathogenesis-related proteins (PRs) in the *Xoo* and *Xoo*+*PP* plants were compared (Figure 4C). In this case, the mRNA levels measured in the *Xoo* plants were used as reference, and compared with the mRNA levels of PRs in the *Xoo*+*PP* plants. It was found that five PRs were significant up-regulated in the *Xoo*+*PP* plants, including *OsPR2*, *OsPR3*, *OsPR6b*, *OsPR8a,* and *OsPR8b*; whereas five PRs, including *OsPR1a*, *OsPR4a*, *OsPR4b*, *OsPR6a,* and *OsPR10*, were significantly down-regulated. These results suggested that the rice plants are inducing the expression of alternative PRs depending on the presence and absence of *P. putida*. *OsPR2*, *OsPR3*, *OsPR6b*, *OsPR8a,* and *OsPR8b* were up-regulated with the accumulation of ABA, revealing a possible link between ABA signaling and an alternative defense pathway.

## 3. Discussion

*OsHDA702* is a histone deacetylase, which belongs to the IAA signaling pathway and is known be directly involved in rice growth [25,26]. The over-expression of *OsHDA702* can lead to increased growth rate and can alter root architecture [27]. The induction of the IAA signaling in plants can be explained considering the observed low ET levels [28]. IAA accumulation in plants led to increase ET synthesis to due to IAA stimulates ACC deaminase activity [29]. It has been reported in several occasions that the release of ACC from the plants into the medium and subsequent reduction of ET levels increases the biosynthesis of IAA and, thus, the overexpression of the IAA signaling pathway, due to the plants try to recover the normal intracellular levels of ACC [17]. In agreement with this mechanism, *OsACC1* and *OsACC3* were significantly up-regulated in the *P. putida*-treated plants (Figure 2A). The up-regulation of *OsHDA702*, together with the up-regulation of *OsACC1* and *OsACC3*, suggest the accumulation of IAA in the rice plants after *P. putida* treatment. Although some bacteria have been reported to produce IAA, *P. putida* KT2440, which was used in this work, does not encode the biosynthesis of IAA, suggesting that the up-regulation of *OsHDA702* might be only related to *P. putida*-induced low ET levels.

Our research group indicated that the release of ACC from rice plants into the medium is produced only during the early rhizobacteria–plant interaction stages; however, the plants stop releasing ACC after this metabolite is accumulated in the medium [19]. Then, the ET levels increase producing a peak of ET that involves the up-regulation of the ET signaling pathway and, subsequently, ISR. Beris et al. observed similar results when detecting the expression of SA and JA-related defense genes in Potato virus Y-infected tomato plants at different time points [20]. It must be noted that the metabolic alterations in response to biotic stress have been usually studied several days after pathogen inoculation when the disease symptoms were obvious, which explains why most reports only indicate the ISR effects. For example, the disease index of *Pectobacterium carotovorum* causing soft rot on *Paenibacillus dendritiformis*-treated potatoes was measured 60 days after inoculation [30]. *Klebsiella* could enhance the ET and JA signaling in peanut plants, inducing ISR against *Aspergillus flavus*; in this case, the relative mRNA levels and lesion length were measured 2 weeks after treatment [31]. In contrast with these results, Yim et al. indicated that *Methylobacterium* spp. reduced the ET levels in tomato plants during 28 days [32]. In that case, the bacterial treatment was repeated once per week, differing from the treatment conditions usually applied in this kind of experiments. In all cases, the ET levels were measured after the bacterial treatment, which could explain why the ET levels were reduced after all measurements.

*OsHDA705* is an ABA-dependent histone deacetylase involved in plant defense [33]. This gene has been related to the defense response of rice plants to abiotic stresses, such as cold [34]. Several reports have demonstrated that rhizobacteria are able to enhance plant resistance to salinity stress, and this effect is produced via up-regulation of ABA signaling [35,36,37,38]. Although the ability of growth-promoting rhizobacteria to induce the accumulation of ABA under abiotic stress has been thoroughly explored, only few reports related to accumulation of ABA in response to pathogen infection in rhizobacteria-treated plants can be found in the literature [39,40]. The antagonistic relationship between ABA and ET signaling is consistent with the observed ABA accumulation and *P. putida*-induced low ET level [41,42]. ABA is known to decrease ET signaling by down-regulating its biosynthesis [29]. It has been reported that exogenous ABA suppresses both JA and ET defense genes, while mutations in the ABA biosynthesis have the opposite effect [43].

**Figure 5:**
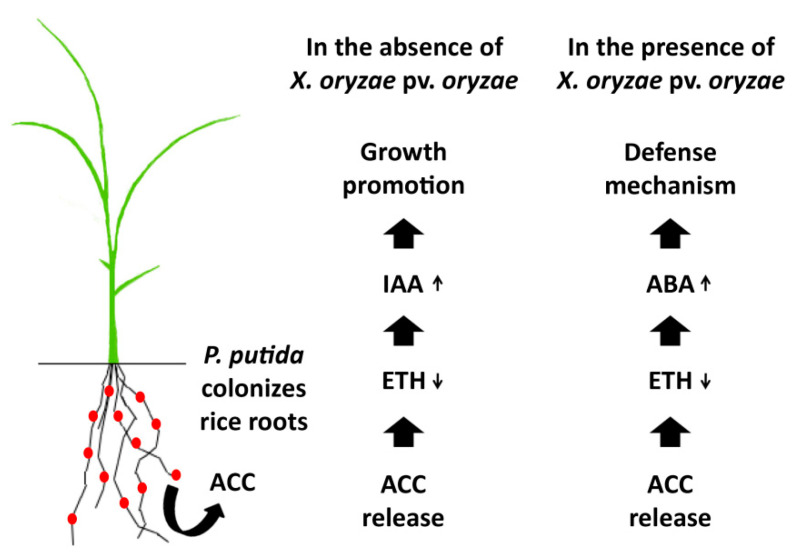
Proposed interaction mechanisms between *P. putida* and rice plants in the presence and in the absence of pathogen infection at the early rhizobacteria–plant interaction stages.

Accumulation of ABA is known to induce a defense response in plants based on the overexpression of antioxidant enzymes. Although the involvement of ABA was not studied, Singh and Jha reported that *Stenotrophomonas maltophilia* enhanced the disease resistance of wheat plants by increasing the activity of β-1,3-glucanase, phenylalanine ammonia lyase (PAL), peroxidase (PO), and polyphenol oxidase (PPO) at the early rhizobacteria–plant interaction stages [44], which seem the typical effects produced via ABA signaling. Similarly, Yim et al. reported that ET production was significantly reduced in *R. solanacearum*-infected tomato plants in the presence of *Methylobacterium* sp., and this effect simultaneously enhanced the expression of phenylalanine ammonia-lyase (PAL) and peroxidases, increasing the disease resistance of the tomato plants against the pathogen [32]. Thus, *P. putida* and other plant growth-promoting rhizobacteria appear to induce a defense response at the early interaction stages that seems more representative for a defense response against abiotic stress than against biotic stress (Figure 5).

Interestingly, it was observed that the metabolic effects produced by *P. putida* are enhancing the overexpression of several PRs. This result is consistent with the accumulation of ABA, which is known to induce the selective up-regulation of some PRs under stress conditions [45,46]. The resistance of rice plants to *X. oryzae* pv. *oryzae* is known to be dependent on the overexpression of *OsPR1a* [47]. As indicated in Figure 4C, *OsPR1a* was down-regulated in the *P. putida*-treated plants, which explains why the metabolic effects are increasing the susceptibility of the rice plants against this pathogen. In contrast, Yim et al. indicated that *Methylobacterium* spp. reduced the ET levels in tomato plants and, at the same time, enhanced the disease resistance of the plants to *Ralstonia solanacearum* [32]. For this reason, we believe that the alternative defense mechanism, possibly based on ABA accumulation, may result in the induction or reduction of disease resistance depending on the pathogen of study, and the specific PRs that are efficient against each pathogen. Although the observed alternative mechanism involved the up-regulation of *OsPR2*, *OsPR3*, *OsPR6b*, *OsPR8a,* and *OsPR8b*, our previous studies of ISR in rice showed the up-regulation of *OsPR1a*, *OsPR1b*, *OsPR5*, *OsPR8c*, *OsPR9,* and *OsPR10* [19], which suggests that the former PRs are not be involved in the ET and JA signaling, whereas the second ones might be related to these hormones.

## 4. Materials and Methods

### 4.1. General Information and Strains

The experiments were carried out using *O. sativa* subsp. *japonica* “Nipponbare” plants. *P. putida* KT2440 and *E. coli* BL21 (DE3) were grown in lysogeny broth (LB) medium (10 g/L tryptone, 5 g/L yeast extract, 10 g/L sodium chloride, pH adjusted at 7.0-7.2) at 37 °C with shaking at 200 rpm. For the inoculation experiments, *X. oryzae* pv. *oryzae* PXO99, the causal agent of rice bacterial blight, was used [47]. This strain was grown nutrient broth (NB) medium (3 g beef extract paste, 1 g yeast extract, 5 g peptone, and 10 g sucrose, pH 7.0-7.2, in 1 L distilled water) at 28 °C with shaking at 200 rpm.

Mass spectrometry analyses were carried out in a QTRAP 5500 Linear Ion Trap Quadrupole MS/MS Mass Spectrometer (AB Sciex Instruments, Framingham, MA, USA).

### 4.2. Cultivation and Treatment of Rice Plants with P. putida

Thirty “Nipponbare” seeds were submerged in a bottle containing 100 mL sterile distilled deionized (DD) water. After 48 h at 37 °C, the water was discarded. Then, 20 germinated seeds were transferred to one pot containing 500 mL aqueous nutrient solution (28.6 mg/L NH_4_NO_3_, 36.7 mg/L CaCl_2_·2H_2_O, 40.5 mg/L MgSO_4_·7H_2_O, 89.3 mg/L K_2_SO_4_, 50.3 mg/L Na_2_HPO_4_·2H_2_O, 11.6 mg/L Na_2_EDTA·2H_2_O, 8.7 mg/L FeSO_4_·7H_2_O, 1.8 mg/L MnCl_2_·4H_2_O, 1.2 mg/L H_3_BO_3_, 9.25 mg/L (NH_4_)_6_MoO_24_·4H_2_O, 43.8 mg/L ZnSO_4_·7H_2_O, and 38.8 mg/L CuSO_4_·5H_2_O). The plants were incubated at 30 °C (day) and 28 °C (night) with a 12 h photoperiod under artificial light in hydroponic cultures (Figure S1). After 7 days, the medium was discarded, and 400 mL of DD water containing *P. putida* were added to the pot. *P. putida* was grown in LB medium (400 mL) to OD_600_ = 1.5. Then, the bacterial cells were washed twice with sterile DD water (50 mL), and suspended in sterile DD water for the plant–bacteria treatment. The concentration of *P. putida* in the treatment solution depended on the specific experiment, and is detailed below for each experiment. The plants were incubated with the bacterial suspension at 30 °C (day) and 28 °C (night) with a 12 h photoperiod until the realization of the experiments.

### 4.3. Microscope Observation of P. putida Colonizing Rice Root

Commercial pCS2^+^-GFP plasmid, containing the enhanced green-fluorescent protein gene (*eGFP*, Gene ID: 20473140), was transformed into the host strains, *P. putida* KT2440 and *E. coli* BL21 (DE3), via electroporation (2.5 kV). *E. coli* BL21 (DE3) was used as negative control. The transformants were selected on LB-agar medium in the presence of ampicillin (50 μg/mL). Rice roots were treated with 10^9^ cells/mL bacterial suspensions. After 24 h, the rice roots were scanned using an inverted microscope (Nikon A1RHD25 inverted microscope).

### 4.4. Measurement of Root and Stem Elongation

The bacterial treatment was carried out using *P. putida* at 0.25, 0.5, 1 and 2 × 10^9^ cells/mL. The control experiment was performed with sterilized DD water in the absence of *P. putida*. For each treatment condition, 3 pots were used, with 20 rice plants in each pot. The stem and root lengths were measured just before the bacterial treatment, and 2 days after the bacterial treatment. The elongation effect was calculated as the difference between both values.

### 4.5. Detection of mRNA Levels of Key Genes in Rice Plants

The bacterial treatment was performed using *P. putida* at 2 × 10^9^ cells/mL; whereas sterilized DD water, in the absence of *P. putida*, was used for the control experiment. The plants were incubated for 2 days after the bacterial treatment. Then, five treated plants and five control plants of similar size were frozen with liquid nitrogen. Total RNA of the five plants was extracted using TRIzol reagent (Invitrogen). The residual DNA was removed using RNase-free DNase I (TaKaRa). Total RNA was extracted using TRIzol reagent (Ambion, Austin, TX, USA). The residual DNA was removed and the first strand cDNA was synthesized in one pot using the Transcript All-in-One First-Strand DNA Synthesis Super Mix for qPCR (One Step gDNA Removal) Kit (Tsingke, Beijing, China). Quantitative real-time PCR was performed using a set of 2 PCR primers with SYBR Green I Real Time PCR (Solarbio, Beijing, China). The PCR analysis was carried out using a 7500 Real time PCR system (Applied Biosystems, Foster City, CA, USA). In all cases, *OsActin* (AK060893) was used as a reference gene, and the relative gene expression was calculated by the 2^−ΔΔCt^ method (Table S1). Three replicates were performed.

### 4.6. Detection and Quantification of ACC in the Culture Medium of Rice Plants

The detection of ACC in the culture medium was examined 0, 1 and 2 days after *P. putida* treatment. The bacteria treatment was performed using *P. putida* at 0.25, 0.5, 1 and 2 × 10^9^ cells/mL. Sterilized DD water, in the absence of *P. putida*, was used for the control experiment. For each treatment condition, 3 replicates were performed (3 pots were used for each treatment conditions with 20 rice plants in each pot).

The detection and quantification of ACC was carried out as previously reported by our research group using the Marfey’s reagent (Figure S2) [19].

### 4.7. Inoculation of X. oryzae pv. oryzae in Rice Plants

The bacterial treatment was performed using *P. putida* at 0.25, 0.5, 1 and 2 × 10^9^ cells/mL; whereas sterilized DD water, in the absence of *P. putida*, was used for the control experiment. For each treatment condition, 3 pots were used, with 20 rice plants in each pot. The inoculation of *X. oryzae* pv. *oryzae* was carried out immediately after the treatment with *P. putida*.

*X. oryzae* pv. *oryzae* was grown in NB medium (50 mL) to OD_600_ = 1.5. The bacterial suspension was centrifuged at 10,000 rpm and 4 °C for 10 min. Then, the cells were washed twice with 10 mL sterile DD water, and suspended in sterile DD water to obtain a solution of OD_600_ = 1. The inoculation of *X. oryzae* pv. *oryzae* was performed using sterile scissors, which were dipped in the bacterial suspension and used to cut the plants at approximately 1 cm from the top of the leaf [48]. Sixty leaves were inoculated (20 leaves from each pot). The length of the lesion caused by *X. oryzae* pv. *oryzae* was measured after 2 days. All inoculated leaves were assessed.

### 4.8. Measurement of ET Levels in Rice Plants

To measure the ET levels, 3 pots with treated plants (2 × 10^9^ cells/mL of *P. putida*) and 3 pots with control plants (with sterilized DD water) were prepared, each pot containing 20 rice plants. ET production levels were measured 2 days after the bacterial treatment. To measure the ET levels, 30 average-sized treated rice plants were selected from the three pots (10 plants from each pot). Each plant was stored in a closed 20 mL vial containing 1 mL sterile DD water for 1 day. Two milliliters of the gas inside the vial were injected into a gas chromatography instrument (Agilent Technologies 7890A GC System). Chromatograms were recorded using an Agilent HP-PLOT-Q capillary column, FID detector and 1 mL/min gas flow rate. Column temperature was maintained as follows: 32 °C from 0 to 1 min, 32 °C to 70 °C from 1 to 2 min, 70 °C from 2 to 3 min, and 70 °C to 160 °C from 3 to 12 min. The injector and detector temperatures were 200 °C and 250 °C, respectively. The peak corresponding to ET appeared at 5.5 min. The ET emission in plants was calculated according to the peak area. Commercial ET was used for calibration between 50 and 0.05 ppb.

### 4.9. Detection and Quantification of ABA in Rice Leaves

Four parallel experiments were performed: (a) Rice plants were treated with *P. putida* (2 × 10^9^ cells/mL) and simultaneously infected with *X. oryzae* pv. *oryzae*; (b) rice plants were treated with *P. putida* (2 × 10^9^ cells/mL) in the absence of pathogen; (c) rice plants were infected with *X. oryzae* pv. *oryzae* in the absence of *P. putida*; (d) rice plants were treated with sterile DD water in the absence of pathogen (control experiment). For each treatment condition, ABA was detected in 20 rice plants (1 pot was used for each treatment condition and each pot contained 20 rice plants). One hundred milligrams of leaf tissue was smashed in an eppendorf tube. Then, 100 µL ethanol was added in each tube, and the resulting suspension was shaken at 10,000 rpm during 5 min. Then, the ethanolic solution was collected. After filtration, 5 µL of solution was injected into the HPLC. HPLC conditions: Reversed phase HPLC (Agilent 1200 Series, USA) at 270 nm using an Eclipse XDB-C18 column (250 × 4.6 mm, Agilent). The mobile phase was 5% to 50% CH_3_CN in H_2_O from 0 to 30 min, and 5% CH_3_CN in H_2_O from 30 to 40 min (column temperature: 30 °C; injection volume: 20 µL). ABA appeared at 25.6 min retention time using the aforementioned conditions (Figure S3). The concentration of ABA in the solutions was calculated according to the peak area using a standard curve from 0 to 0.05 mg/mL ABA. The peak corresponding to ABA was submitted to MS spectrometry analysis, proving an *m*/*z* peak at 263.1, which was in agreement with the expected molecular weight, [M-H]^−^ = 263.1283.

### 4.10. Data Analysis

The statistical analyses were performed using SPSS (Statistical Package, v. 20.0). The different variables were subjected to Student’s *t*-test and were tested for significant differences at the *p* < 0.05 (*), *p* < 0.01 (**), *p* < 0.001 (***), and *p* < 0.0001 (****) levels (ns = no significance). The standard deviation calculated using Microsoft Excel 2010 was used to quantify the dispersion. For the root and stem elongation effect, lesion length and ET emission experiments, all values recorded in the three pots with the same treatment conditions were pooled and used to calculate the standard deviation.

## Supplementary Materials

The following are available online at https://www.mdpi.com/2223-7747/9/12/1641/s1, Table S1: Primers used in qRT-PCR, Figure S1: Cultivation of *O. sativa* ‘Nipponbare’ plants in pots with 0 and 2 × 10^9^ cells/mL *P. putida*, Figure S2: HPLC-based analysis of labelled ACC in the medium of *P. putida* treated rice plants, Figure S3: HPLC-based analysis of ABA in rice leaves.
