# Supplementary material for: Pseudomonas putida Represses JA- and SA-Mediated Defense Pathways in Rice and Promotes an Alternative Defense Mechanism Possibly through ABA Signaling
Source: Plants (Basel). 2020 Nov 24;9(12):1641. doi: 10.3390/plants9121641 (PMC7760693; doi:10.3390/plants9121641)
Supplement: Supplementary file 1 [file plants-09-01641-s001.pdf]

Supplementary Material

Pseudomonas putida Represses JA- and SA-Mediated Defense Pathways in Rice and Promotes an Alternative Defense Mechanism Possibly through ABA Signaling

Rui Wang [1,#], Hai-Lin Wang [1,#], Rui-Ping Tang [1], Meng-Ying Sun [1], Tang-Min Chen [1], Xu-Chu Duan [1], Xiao-Feng Lu [1], Dong Liu [1], Xin-Chi Shi [1,*], Pedro Laborda [1,*] and Su-Yan Wang [1,*]

School of Life Sciences, Nantong University, Nantong 226019, People's Republic of China

[#]These authors contributed equally.

*Corresponding authors:

Dr. Suyan Wang; email: wangsuyan@ntu.edu.cn

Prof. Pedro Laborda; email: pedro@ntu.edu.cn

Dr. Xinchi Shi; email: shxch0301@ntu.edu.cn

Table S1. Primers used in qRT-PCR.

Gene	RGAP number	Primer_F(5´ - 3´)	Primer_R(5´ - 3´)
OsACC1	Os03g0727600	GTGTCGTCCCAGACGCAGTA	TCCACCCAGCAGAAGAGGC
OsACC3	Os05g0196600	GGGTTTGACAGGGACTTGA	GTTCGACGGGTTCGTGAT
OsPR1a	Os01g0382000	TGGGTGTCGGAGAAGCAGTG	GGTGATGAAGACGCCGAGG
OsPR1b	Os07g0129200	ACGCCTTCACGGTCCATAC	AAACAGAAAGAAACAGAGGGAGTAC
OsPR2	Os01g0940700	TTGCGGCCATTCCTACAGT	TGGTGAGGGCGATGCTTG
OsPR3	Os04g0493400	ACCGCACCAACGACGACT	TGTCTGGGTCCCTTAGCC
OsPR4a	Os11g0592200	GCAAGTGTATCCAGGTGAAGAA	ACCCATCTGGTAGCCACGAC
OsPR4b	Os11g0592100	TGGATGGACCGCCTTCTGT	CGTCGCTGTCGATCTTGGAG
OsPR5	Os12g0628600	CTGACGCTGGCGGAGTTC	CTTGGTGTTGTCTTCGGGGA
OsPR6a	Os01g0124650	GCAGCCGCTCGATGTTGT	AAGATGAAGACCGCCATGACTA
OsPR6b	Os01g0803200	GAACGAGAACGACCTCCACC	AGTACAAAGTGCCAGCGACAAC
OsPR8a	Os02g0771700	CCCGTTCTTCGCGTACTCTG	GTCTCCTTGGCGTCACCCT
OsPR8b	Os10g0416500	ACCCGAACATCGCCACC	CCTGCTCGTCGTAGAACATCA
OsPR8c	Os01g0860500	ACCAGGTCGCTCGTCTCCA	AACCCGCCGTGCCAGTA
OsPR9	Os07g0677200	GGCAAATACCGACCTCCCT	TCGTTGTAGATCCTGTCCCTGA
OsPR10	Os12g0555500	ACACTCGACGGAGACGAAGC	CAGGGTGAGCGACGAGGTA
OsNPR1	Os01g0194300	TTTCCGATGGAGGCAAGAG	GCTGTCATCCGAGCTAAGTGTT
OsACO	Os01g0580500	TCAATGGCTACCACGTTAGATG	GATGTGACAGCCAAGAATTTCA
OsLOX	Os03g0738600	GCATCCCCAACAGCACATC	AATAAAGATTTGGGAGTGACATA
OsMPK6	Os10g0533600	ATGGGTTCCGTCCTGAAGGC	CACTGAGCTGATAGGGTCGC
OsGAMYB	Os01g0812000	GGGATCTGGAGAGCAAGTAATG	GGATCAGCTGTATTCCCAGAAA
OsYAB4	Os02g0643200	TTTCCCTCACATCCATTTTGG	AGAACCATCCTGAGGCTTGAAG
OsABI5	Os01g0859300	ACACACCGGCCAATCGAT	AGCGGGAAACACAAAGTGAAG
OsHDA702	Os06t0583400	TTGAGGTATCTGCCGTAGTC	CATGGTTTCGACGTGTTCTA
OsHDA705	Os08g0344100	GCATTGATGATGATACCTTTCGTG	GGCTGAGAAACCGACCTCTG
OsActin	Os03g0718100	ATGCTATCCCTCGTCTCGAC	CGCACTTCATGATGGAGTTG

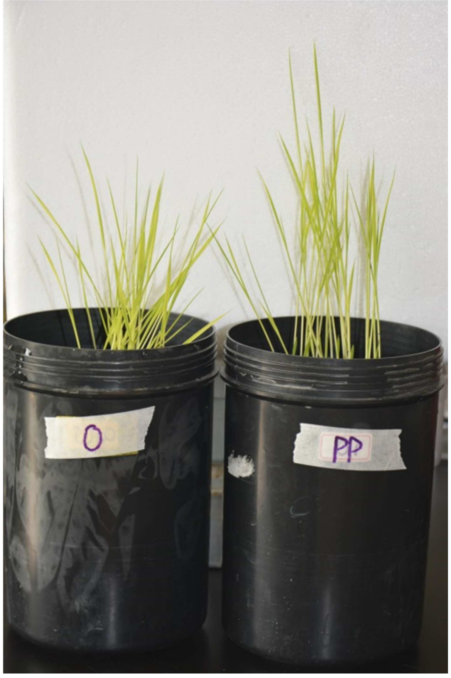

Figure S1. Cultivation of *O. sativa* 'Nipponbare' plants in pots with 0 and 2×10^9 cells/mL *P. putida*. The images were taken 2 days after the bacterial treatment.

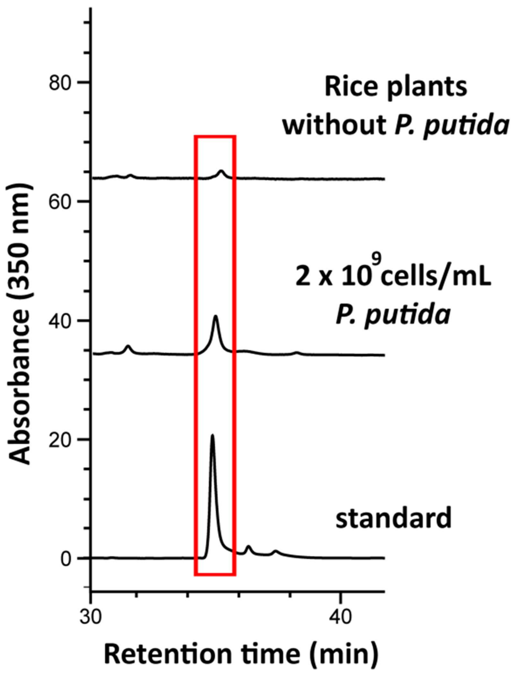

Figure S2. HPLC-based analysis of labelled ACC in the medium of *P. putida* treated rice plants. The labelling was carried out using the Marfey's reagent.

HPLC conditions: reverse phase HPLC (Agilent 1200 Series, United States) at 350 nm using an Eclipse XDB-C18 column (250 × 4.6 mm, Agilent) at 35 °C. The mobile phase was 0% to 60% CH_3CN in H_2O from 0 to 60 min, 90% CH_3CN in H_2O from 60 to 70 min, and 0% CH_3CN in H_2O from 70 to 75 min (column temperature: 30 ºC; injection volume: 5 μL).

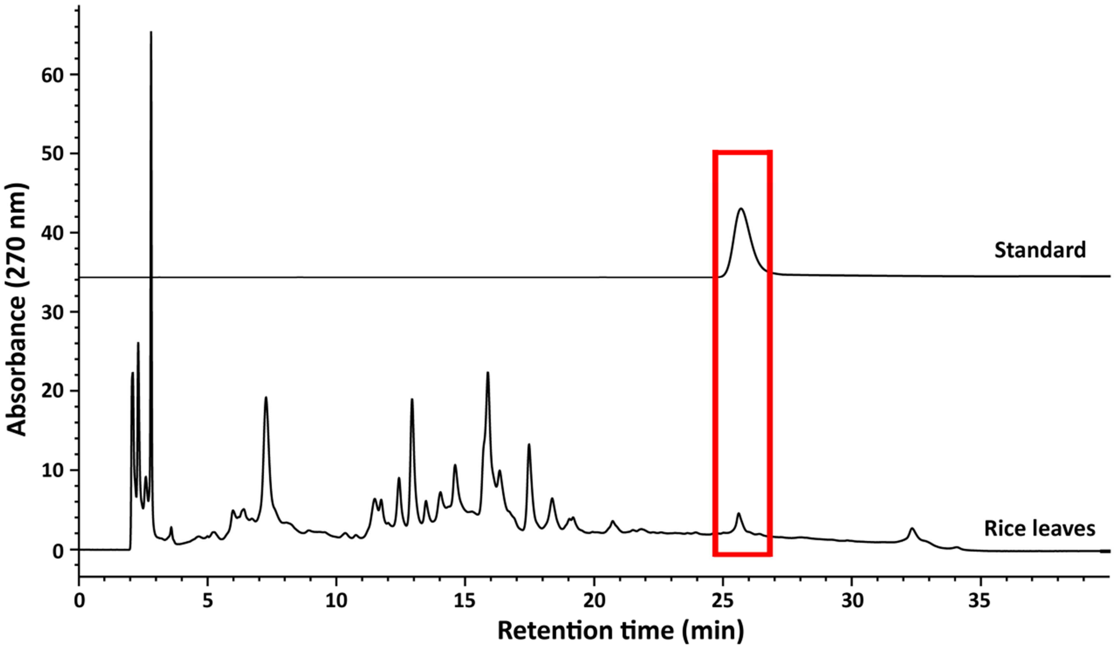

Figure S3. HPLC-based analysis of ABA in rice leaves.

HPLC conditions: reversed phase HPLC (Agilent 1200 Series, United States) at 270 nm using an Eclipse XDB-C18 column (250 × 4.6 mm, Agilent). The mobile phase was 5% to 50% CH_3CN in H_2O from 0 to 30 min, and 5% CH_3CN in H_2O from 30 to 40 min (column temperature: 30 ºC; injection volume: 20 μL).
